# Genetic patterns in *Mugil cephalus* and implications for fisheries and aquaculture management

**DOI:** 10.1038/s41598-021-82515-7

**Published:** 2021-02-03

**Authors:** Piero Cossu, Laura Mura, Fabio Scarpa, Tiziana Lai, Daria Sanna, Ilenia Azzena, Nicola Fois, Marco Casu

**Affiliations:** 1 Department of Sciences for Nature and Environmental Resources, University of Sassari, Sassari, Italy; 2 Department of Veterinary Medicine, University of Sassari, Sassari, SS Italy; 3 Dipartimento per la Ricerca Nelle Produzioni Animali, Agris Sardegna, Olmedo, SS Italy; 4 Department of Biomedical Sciences, University of Sassari, Sassari, Italy

**Keywords:** Marine biology, Conservation biology, Molecular ecology, Population dynamics, Ecology, Ecology, Conservation biology, Molecular ecology, Population dynamics, Genetic markers, Population genetics

## Abstract

Exploitation of fisheries and aquaculture practices are exposing marine fish populations to increasing genetic risks. Therefore, the integration of genetic information into fisheries and aquaculture management is becoming crucial to ensure species’ long-term persistence. The raising commercial value of grey mullet (*Mugil cephalus*) and its roe represents a growing challenge to the sustainable management of this economically important fishery resource. Here, microsatellites were used to investigate patterns of genetic variation in a Mediterranean area that harbor flourishing fisheries and practice semi-intensive farming of grey mullet. Genetic diversity within populations is smaller than values reported in previous studies as a result of the lower polymorphism displayed by the new microsatellite loci. Lack of genetic structuring points to the existence of a unique genetic stock, which is consistent with the species’ high dispersal capabilities. Nonetheless, differences in local population effective size as well as the excess of related individuals do not completely fit the picture of a large panmictic population. Baseline genetic information here gathered will allow to set up the genetic monitoring of regional fish stocks, which is needed to assess the impact of both harvesting and aquaculture on the genetic integrity of *Mugil cephalus* wild populations.

## Introduction

The increase of fish consumption during the last decades raised concerns about the overexploitation of marine resources^1^. The need to avoid the excessive erosion of fish stocks boosted research in aquaculture, whose production nowadays is comparable to overall captures^2^. Aquaculture fulfils different goals, one of which aims to replenish or just increase the biomass of wild stocks by sea ranching, stock restoration and stock enhancement^3^. The supplementation of wild stocks with early-generation captive fish may induce a reduction of total population effective size in the supplemented wild population if they are the offspring of a handful of breeders^4^. This artificial bottleneck, known as the Ryman–Laikre effect^5^ may thus lead to the loss of genetic diversity in the combined captive-wild system by increasing inbreeding and random genetic drift^6^. The negative effects of supplementation will be enhanced in marine species subject to variance in reproductive success (recruitment sweepstake effect^7^): in these cases, even a limited reduction of population size can result in the loss of low-frequency alleles that can be important for the adaptation to environmental changes^3^. Therefore, protection of genetic diversity within- and amongst-population should deserve high priority in the planning and implementation of supplementation programmes to preserve species’ adaptive and evolutionary potential and thus their long-term persistence^3^.

The grey flathead mullet (*Mugil cephalus* L.), also commonly referred to as the striped mullet (and henceforth the grey mullet), is a world-wide distributed coastal fish species that spawns offshore and uses estuarine and brackish-water environments as nursery habitats^8^. Early larval stages passively disperse by drifting in ocean currents and then move onshore at the post-flexion larval stage, where they inhabit the surf zone as schools of early juveniles^9^. After about a month spent in the sea, fry colonises coastal ponds, lagoons, estuaries and sometimes the adjoining river catchments^10^. Adult individuals migrate back to sea, where they show highly variable dispersal capabilities, whose extent may range from 32 to 700 km^9^. The species is an important economical resource worldwide and in Mediterranean waters^9^, where it is captured during the spawning migration to harvest the egg roe, which is salted and dried to be sold as a delicatessen^11^. In many parts of the World, the increased demand for mullet roe raised the species’ commercial value, which has been called ‘‘the grey gold’’ by fishermen^9^. Therefore, the grey mullet is often stocked in brackish coastal lagoons to improve fish yield, raised in commercial freshwater ponds, and it has also been introduced into inland freshwater lakes and reservoirs to develop new fisheries^9^.

Notwithstanding the growing interest in fisheries and aquaculture of *M. cephalus*, few studies investigated the distribution of genetic diversity at the spatial and temporal scales that are relevant to the management of this natural resource. Perhaps due to its high dispersal potential, genetic studies focussed mainly on investigating large-scale genetic structuring and assessing whether *M. cephalus* is a single, cosmopolitan species or a complex of cryptic species (e.g.^12–15^. Consistent with this picture, an overall lack of genetic structuring was reported at regional and basin-wide spatial scales^16,17^. However, the use of more variable markers as microsatellites evidenced genetic structuring within the Mediterranean Sea, roughly matching well-known biogeographical barriers to dispersal, as well as an isolation by distance pattern^18^.

**Figure 1:**
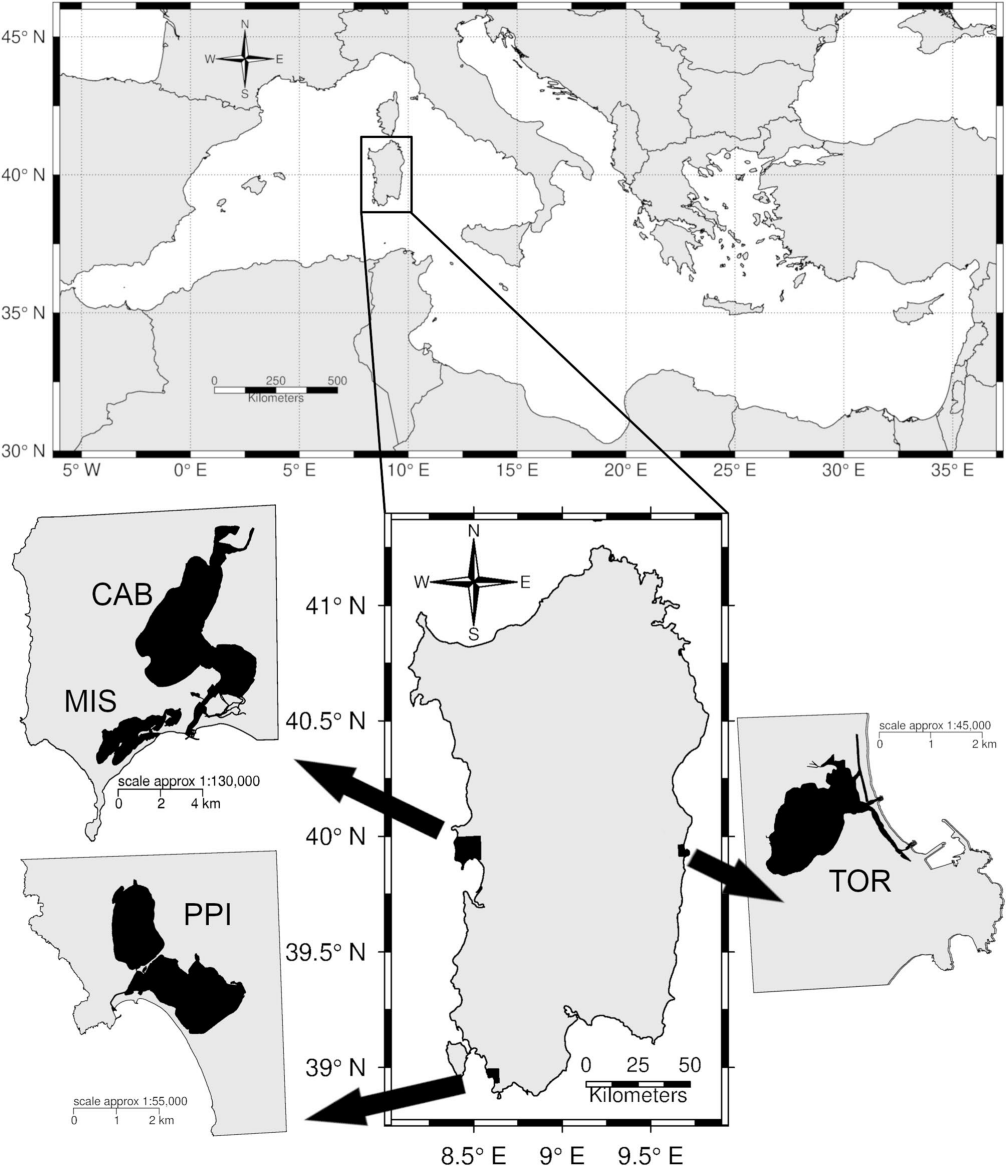
Distribution map of the sampling areas. Note the different scale among coastal ponds. *CAB* Cabras pond, *MIS* Mistras pond, *PPI* Porto Pino ponds, *TOR* Tortolì pond. This map was created using R 3.5.2 (https://www.r-project.org/) and then modified using the free software GIMP 2.10.2 (https://www.gimp.org/).

Therefore, one knowledge gap to be filled concerns the distribution of genetic diversity on local and regional scales in those areas where the grey mullet is, or can be, a commercially valuable resource for fisheries and aquaculture. This is one of the main goals envisioned in the responsible genetic approach to fisheries and aquaculture practises: the protection of the genetic integrity of wild populations^3^. The distribution of genetic diversity in wild populations represents the baseline information to plan and implement management strategies that minimise genetic risks for natural populations, thus preserving the long-term species persistence^19^. Here, we focus our attention on several populations of *M. cephalus* from Sardinia island (Fig. 1), where the species represents an important economic resource: in 2017, 156 out of 401 tonnes of total captures from Italy were indeed from Sardinian coastal waters^2^. Furthermore, to increase or maintain yields over years, mullets are cultured in semi-intensive, extensive systems either to be marketed for direct human consumption or to harvest mullet roe^11^. However, in recent years the amount of fish captured by fishermen covered only a small part of the growing market demand for mullet roe, forcing Sardinian manufacturers to purchase an increasing amount of frozen egg roe from fishing areas other than the Mediterranean Sea^20^.

The aim of this study was to investigate patterns of genetic diversity in exploited populations of *M. cephalus*, which will provide the baseline information needed to enforce a responsible genetic approach for this commercially valuable resource. Genetic data will help to plan a more effective fishery management, as well as sea ranching or restocking programmes that minimise potential genetic risks arising from the inter-breeding amongst wild and farmed individuals. To achieve this goal, we set up multiplex PCR reactions based on available microsatellite loci for this species: these markers are a suitable tool to carry out regional- and fine-scale population genetic studies in the grey mullet^21^.

## Results

### Marker validation

All microsatellite loci were polymorphic at the 5% level across all populations (Supplementary Table S3), with the number of alleles ranging from 3 at locus Mce11 to 32 at locus Mcs2DM (Supplementary Table S4). Overall, 7 out of 14 loci departed from HWP: 5 loci showed a heterozygote deficit in at least one population (Mce22, Mce6, Mce24, Mce25 and Mce3), whereas Mcs16DM and Mcs2FH displayed a heterozygote excess at MIS and PPI, respectively. Based on the LM method, two loci (Mce25 and Mce3) displayed a significant departure from HWP (*P* < 0.05), which cannot be explained by chance alone. The presence of null alleles was the most likely reason for the heterozygote deficit observed at Mce25 and Mce3 (Supplementary Table S5): as their frequency exceeded the threshold of 0.08 in both detection methods, these loci were excluded from downstream analyses. Another locus, Mce22 showed null allele frequencies > 0.08 at MIS and was thus removed from the final dataset.

Overall, 96 out of 364 locus pairs showed LD, which largely exceeded those expected by chance, based on a cumulative binomial distribution (27 locus pairs, *P* = 0.05). The locus pairs showing LD were unevenly distributed across populations: 8, 70, 10 and 8 pairs at CAB, MIS, PPI and TOR, respectively. After applying the FDR correction, the number of locus pairs showing LD was nearly halved (58), but still 54 pairs were found in MIS versus one or two pairs in the other populations, thus suggesting that population level processes such as tiny population size might be responsible for high levels of apparent LD in this population. Overall, 15 locus pairs showed LD in at least two populations, evidencing significant departures according to the LM method (*P* < 0.05). However, most of such locus pairs involved loci that did not show LD in former studies^18,21^; therefore, it seems unlikely that LD was due to true non-random associations amongst the same loci in the present study. Nevertheless, to be as conservative as possible, one further locus (Mcs16DM) that showed LD with locus Mcs1EH in all populations was cautiously excluded from downstream analyses. Finally, no locus was detected as a candidate for selection by all the outlier detection methods simultaneously (Supplementary Figs. S1, S2 and S3). The locus Mce24 was detected as a potential outlier for balancing selection by the Bayesian method but not the other two methods (Supplementary Fig. S1). In contrast, the *LnRH* statistic detected the loci Mcs2DM and Mcs15CM as potential candidates for balancing selection at 95% level but they lie within the expected distribution for neutral alleles after applying the FDR correction (Supplementary Fig. S2). Instead, no locus was detected as an outlier for either divergent or balancing selection by the FDIST2 approach (Supplementary Fig. S3). Therefore, considering loci detected by only one method as false positives, all loci were subsequently deemed as selectively neutral. Based on these results, 10 out 14 loci were retained for downstream analyses.

### Genetic diversity and population effective size

The number of alleles (*A*), the allelic richness (*A*_R_), the mean observed and expected heterozygosity (*H*_O_ and *H*_E_, respectively), and the inbreeding coefficient (*F*_IS_) are summarised in Table 1. Values of *H*_E_ were similar across samples with the highest value found in PPI (mean *H*_E_ = 0.81 ± 0.02); this population also showed the largest allelic richness (*A*_R_ = 13.66 ± 0.87). Nonetheless, pairwise Wilcoxon rank-sum tests did not evidence differences amongst any population pair at both metrics of genetic diversity within populations (*P* > 0.05).

**Table 1:** Summary statistics of within population genetic variation averaged over loci for each population.

	*N*	*A* ± SE	*A*_R_ ± SE	*H*_E_ ± SE	*H*_O_ ± SE	*F*_IS_ (95% CI)
CAB	52	15.60 ± 0.85	12.64 ± 0.70	0.79 ± 0.02	0.78 ± 0.02	− 0.006 (− 0.039, 0.028)
MIS	34	14.10 ± 1.02	12.14 ± 0.85	0.79 ± 0.03	0.81 ± 0.03	− 0.026 (− 0.075, 0.022)
PPI	48	16.90 ± 1.09	13.66 ± 0.87	0.81 ± 0.02	0.83 ± 0.02	− 0.031 (− 0.050, − 0.006)
TOR	50	15.60 ± 0.89	12.83 ± 0.73	0.79 ± 0.03	0.79 ± 0.03	0.003 (− 0.031, 0.037)

CAB, Cabras pond; MIS, Mistras pond; PPI, Porto Pino ponds; TOR, Tortolì pond; *N*, sample size; *A*, number of alleles; *A*_R_, allelic richness; *H*_E_, expected heterozygosity; *H*_O_, observed heterozygosity; *F*_IS_, inbreeding coefficient; SE, Standard Error; 95% CI, 95% Confidence Interval.

The heterozygosity excess test did not evidence signatures of recent population declines (Wilcoxon sign-rank test, *P* > 0.05): results were not affected by varying model parameters, thus showing to be robust to different model assumptions (Table 2). All populations displayed finite mean estimates of contemporary population effective size, albeit only one population (MIS) also showed a finite *N*_e_ estimate for the upper bound of the 95% CI (Table 2). The latter population was characterised by the smallest *N*_e_ estimate and the 95% CI of population effective size did not overlap with those of the other populations. Remarkably, CAB, which is the geographically closest population to MIS showed the largest mean population effective size (*N*_e_ = 756) with a lower bound (*N*_e_ = 131) that is more than three times larger than the upper bound estimated for the latter (*N*_e_ = 35).

**Table 2:** Mean current effective population size and Heterozygosity excess test for population size decline.

	Population effective size	Heterozygosity excess test		
	*N*_*e*_^˄^ LD	*P*_TPM70_	*P*_TPM80_	*P*_TPM90_
CAB	756 (131–∞)	0.91	0.95	0.99
MIS	17 (9–35)	0.96	0.99	0.99
PPI	289 (93–∞)	0.84	0.93	0.98
TOR	554 (158–∞)	0.69	0.88	0.90

Contemporary population effective size was estimated using the Linkage Disequilibrium method (*N*_e_^˄^ LD); 95% confidence interval (CI) of *N*_e_ based on Jackknifing over loci is reported within brackets. Recent population bottlenecks were assessed testing for the probability of heterozygosity excess related to the heterozygosity expected at mutation-drift equilibrium (*H*_E_ > *H*_EQ_). Equilibrium heterozygosity was estimated by a Two-Phase Mutation Model with increasing proportion of single-step mutations: 70% (*P*_TPM70_), 80% (*P*_TPM80_) and 90% (*P*_TPM90_). Populations are abbreviated as in Table 1.

### Genetic structure and kinship analysis

Since results based on *D*_EST_ values (Supplementary Table S6) mirrored those resulting from *F*_ST_, only the latter are reported hereafter. Global *F*_ST_ did not show significant genetic differentiation amongst populations as its confidence interval straddled zero (*F*_ST_ = − 0.0002; CI_95_ = − 0.004, 0.004). Consistent with this picture, the exact G test of population differentiation did not evidence heterogeneous genotypic frequencies amongst samples (*χ*^2^ = 29.06 with 20 degrees of freedom, *P* = 0.086). Nor pairwise *F*_ST_ values nor exact G tests after correction for multiple testing (FDR method) evidenced genetic divergence amongst any population pair (Table 3). The simulation of pseudo-datasets with different levels of population differentiation indicated that the actual dataset reached almost 95% power to detect genetic divergence when *F*_ST_ = 0.0025 (Supplementary Fig. S4). When *F*_ST_ ≥ 0.005, the power of detecting population differentiation was perfect; moreover, dataset was unlikely to infer genetic heterogeneity when it is not true nor was too conservative (*α*-error = 0.024).

**Table 3:** Pairwise population differentiation estimated using Weir and Cockerham’s *ϴ*.

	CAB	MIS	PPI	TOR
CAB	–	(− 0.0083, 0.0065)	(− 0.0048, 0.0066)	(− 0.0047, 0.0073)
MIS	− 0.0021	–	(− 0.0074, 0.0079)	(− 0.0067, 0.0095)
PPI	0.0003	− 0.0007	–	(− 0.0057, 0.0071)
TOR	0.0006	0.0003	− 0.0001	–

Observed values are reported below the diagonal. Lower and upper limits of 95% confidence intervals based on 10,000 bootstraps are reported within brackets above the diagonal. Values outlined in bold indicate population pairs showing significant genetic divergence based on the Fisher’s exact test for genetic homogeneity of genotype frequencies. Due to multiple testing, probability values were adjusted using the False Discovery Rate (FDR) method^52^. Populations are abbreviated as in Table 1.

Bayesian clustering clearly supported *K* = 1 as the best clustering solution (Supplementary Table S7). Given that Bayesian clustering methods perform poorly when *F*_ST_ ≤ 0.01 regardless the number of markers used^22^, we cannot rule out that this outcome might reflect lack of resolving power rather than true panmixia. Using ponds as predefined groups in the Discriminant Analysis of Prinicipal Components (DAPC), 70 principal components were retained based on the cross-validation procedure. The ordination plot of the first two discriminant functions did not highlight a stark separation among individuals from different ponds (Supplementary Fig. S5). Notwithstanding DAPC try to minimize differences within groups, individuals sampled in the same pond were as scattered as, or more scattered than, those from different ponds.

**Figure 2:**
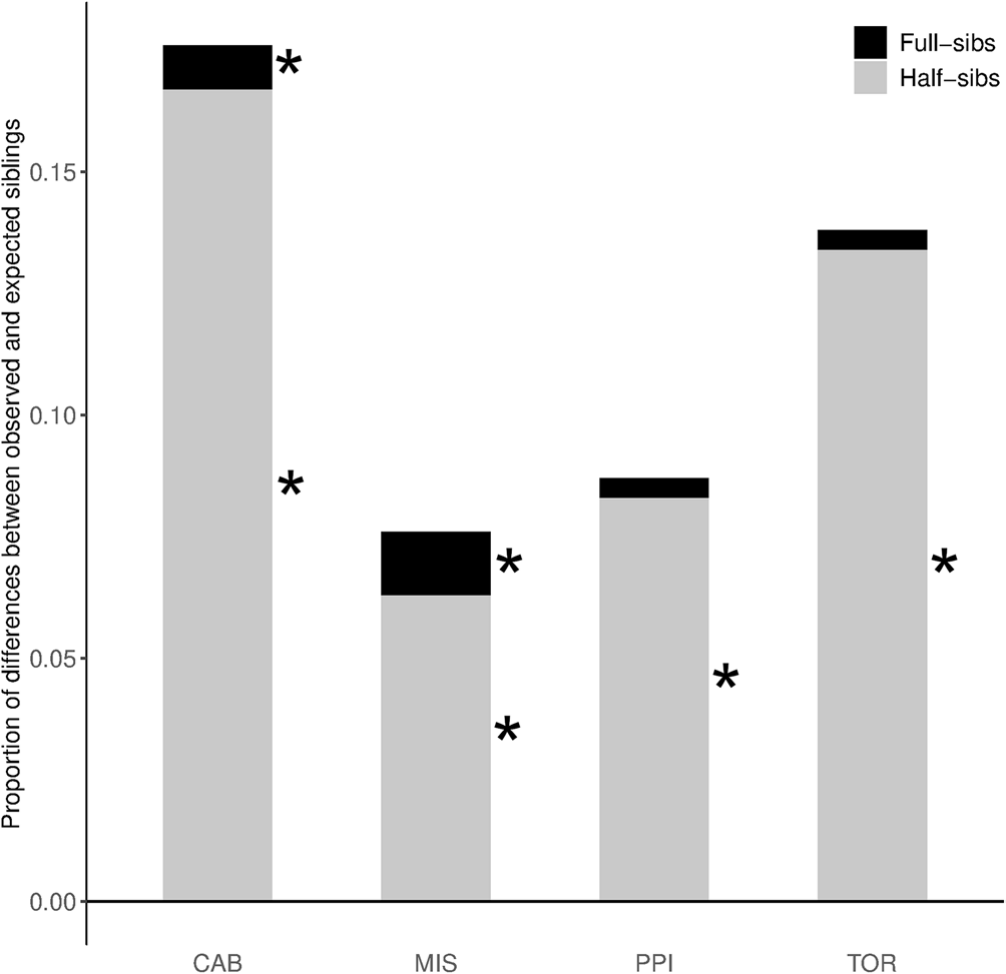
Difference between expected and observed proportions of full- and half-siblings, represented by black and grey bars, respectively. Asterisks mark differences that are significantly larger than those expected by chance. This figure was created using R 3.5.2 (https://www.r-project.org/) and then modified using the free software GIMP 2.10.2 (https://www.gimp.org/).

All the populations considered in the present study showed an overabundance of half-siblings (Fig. 2). The largest difference between observed and expected half-siblings was observed at CAB, which also displayed a proportion of full-siblings larger than expected. Though the excess of half-siblings at MIS was smaller than that observed in other populations, nevertheless the former showed a larger excess of full-siblings than CAB (Fig. 2). Nevertheless, the genotype sharing method showed an overabundance of related individuals compared with those observed using the maximum likelihood (ML) pairwise estimator (Supplementary Table S8). Whilst the proportion of full-siblings detected by both methods was similar in all populations but CAB, the genotype sharing method detected 4–5 times more half-siblings than the ML method.

## Discussion

In the present study, microsatellite data were used to gather the baseline genetic information that is necessary for applying a responsible genetic approach^3^ to fisheries and aquaculture-based management of *Mugil cephalus*. This approach will help preserve the genetic integrity of natural populations according to both FAO^23^ and the Convention on Biological Diversity (Aichi 2010, http://www.cbd.int/sp/) guidelines. It will also complement management strategies aimed at ensuring the sustainable exploitation of this commercially valuable resource.

Overall, patterns of genetic diversity are consistent with former studies^17,18^, even though observed heterozygosity is on average smaller in the present study (Table 1). Such discrepancy reflects the lower polymorphism of some microsatellite loci used in the present study, which are less variable than those developed by Miggiano et al.^21^, rather than small population size or recent population declines (Table 2). In fact, if we consider only the latter markers (Supplementary Table S4), levels of Genetic diversity are as high as those found by the previous studies. By and large, levels of genetic diversity here reported are within the range observed in other species of mullets^14,24,25^.

Based on simulations, power of detecting genetic differentiation is very close to 95% when *F*_ST_ = 0.0025 (Supplementary Fig. S4), which is four times smaller than the threshold that is usually adopted for genetic stock identification (*F*_ST_ = 0.01,^26,27^). Therefore, the panel of microsatellites is suitable to detect population units relevant to fishery management. Accordingly, the lack of genetic differentiation observed amongst the four populations (Table 3), the Bayesian clustering and the DAPC outcomes (Supplementary Table S7 and Figure S5) indicate that the four samples analysed in the present study may belong to a unique genetic stock. This result is consistent with Durand et al.’s^18^ findings: in the Mediterranean, genetic divergence was found across major oceanographic fronts, whereas populations were genetically homogeneous within basins. These results are also consistent with the species’ high dispersal capabilities: migrations of more than 200 km have been observed and adult grey mullets may disperse as far as 700 km^9^. Such migrations may ensure high levels of gene flow amongst locations, leading to genetic homogenisation at regional scales, as observed along the Tunisian coasts^17^.

Nevertheless, some outcomes of the present study do not fulfil completely the picture of a unique large panmictic population. In this case, local effective population sizes are expected to be roughly of the same magnitude as they shall tend to converge on the global metapopulation size^28^. In contrast, the four ponds here studied show different local effective population sizes (Table 2), albeit the large and overlapping confidence intervals do not rule out a similar, large contemporary *N*_e_ in three of them. Mistras lagoon is a noteworthy exception to this trend, as it shows the lowest effective population size amongst all populations, even when accounting for uncertainty. Remarkably, populations from Mistras and Cabras show the largest difference in *N*_e_, albeit the channels connecting the ponds to the sea are only one km apart. Thus, it seems unlikely that the low *N*_e_ recorded at Mistras might reflect recruitment from a larval pool other than Cabras. Small *N*_e_ may depend on recent population declines, which may occur in unpredictable and highly variable environments as brackish-water habitats. Nevertheless, the excess heterozygosity test does not evidence any recent population size decline in Mistras as well as any other population (Table 2). Perhaps, this result depends on the fact that changes in trophic conditions likely affect recruitment of mullets from the Sea^29^ rather than determining post-recruitment decreases of population abundance. Consistent with this picture, Mistras does not show a reduction of genetic diversity relative to other populations, notwithstanding its small *N*_e_. The loss of genetic diversity because of small local effective population sizes may be counterbalanced by dispersal and gene flow: the offshore spawning migrations of adult mullets may increase the global effective population size, thus preventing inbreeding and genetic drift on local scales in the short- and the long-term, respectively^30^.

We may advance two hypotheses that might explain the small local *N*_e_ observed at Mistras compared to the other populations. Genotype-based habitat selection may drive recruitment in fish that use brackish-water environments as nursery habitats: for instance, the white sea bream (*Diplodus sargus*) and the gilthead sea bream (*Sparus aurata*) migrate into those coastal lagoons where juveniles growth is maximised^31–33^. Likewise, larvae and juveniles of *M. cephalus* prefer to settle in oligohaline and mesohaline brackish waters, which favour their growth^34^. Therefore, Cabras likely represents a more suitable habitat than Mistras for the settlement of grey mullet recruits: at the onset of winter season, when juveniles migrate into lagoons, Cabras pond is characterised by mesohaline conditions, whereas Mistras lagoon remains hyperhaline throughout the entire year. We may speculate that early life stages of grey mullet could be mixtures of individuals with different phenotypic traits, each of which maximises growth in different habitats. In this case, different local population sizes will depend on genotype-based habitat selection and the frequency of those genotypes-phenotypes in the global metapopulation rather than sweepstake recruitment events. Otolith microchemistry provides a further line of evidence that may support this hypothesis: in the Mediterraneans Sea, grey mullets show different environmental migratory patterns, spending either part or their entire lifetime in fresh- or brackish-water habitats, as well as preferring seawater and high salinity habitats^35^.

Alternatively, local *N*_e_ might be influenced by the semi-intensive farming of grey mullets, which is a common practise across Mediterranean coastal ponds and in many Sardinian wetlands^11^. The release of such early-generation hatchery fish may trigger the Ryman–Laikre effect in the supplemented wild population if the captive fishes are the offspring of a limited number of breeders^4,8^. In addition to decrease *N*_e_, this process should also result in larger than expected relatedness as significant kinship structure should not occur when effective population size is large^31^. Unexpectedly, all four samples show an excess of half-and/or full-siblings as detected by the genotype sharing method (Fig. 2), albeit this result should be interpreted with caution because of the difficulty to assess relationships among individuals using pairwise relatedness^36^. Indeed, this method estimated a larger proportion of related individuals than that obtained using a maximum likelihood approach and both methods mainly depart in the number of detected half-siblings (Table S8), which likely correspond to the increasing variance of estimators as relatedness decreases^37^. Disregarding the reliability and precision of each method, the proportion of related individuals is similar across all populations (Table S8), hence downplaying the possibility that the small *N*_e_ recorded in Mistras might depend on the Ryman–Laikre effect.

The results outlined above may help complement fishery and aquaculture management in several ways. In the first place, the presence of a unique genetic stock indicates that *M. cephalus* could be considered as a single management unit at regional level, based on the concept that genetically distinct stocks need to be managed as separate units^38^. Second, regional genetic homogeneity will ease the enforcement of a responsible approach to aquaculture-based fisheries management^39^, which may help local small fisheries to withstand yield reductions triggered by environmental fluctuations. Our results suggest that early-generation captive individuals from other ponds could be used to supplement depleted populations without affecting their genetic integrity, unless they are the offspring of a small number of breeders. In this context, the genetic information here gathered is also a crucial prerequisite to select and determine the number of individuals that are needed to raise broodstocks that maintain levels of genetic diversity as high as those of wild populations.

Our results may also provide some clue in on the assessment of demographic independence, which is a goal of great relevance in fisheries management. Although it is difficult to delineate demographic units with genetic data^27,40^, kinship analyses may help reach this goal as these metrics are suitable to assess ongoing dispersal and thus local recruitment patterns^41^. Nevertheless, caution is needed to avoid a biased estimation of these demographic processes as the present study further evidences the difficulty of estimating relatedness among individuals^37^. Based on our results (Supplementary Table S8), considering only high-level relationships might reduce the uncertainty in the estimation of relatedness, which is analogous to increase the threshold above which individuals are deemed as related^42^. Alternatively, only relationships that overlap across two or more methods could be deemed as reliable; in this case, however, recruitment patterns will be determined by the most conservative method or that underestimating relatedness.

In conclusion, our results further confirm the importance of using several lines of evidence to draw inferences on species’ fine-scale genetic patterns. This framework suits the needs of fishery managers, who usually adopt a ‘best available science’ approach in which information from several sources and the associated uncertainty are considered before making management decisions^40^. For instance, local population effective size clues in on a factor that may hamper the supplementation of wild populations. Habitat selection might dampen the success of aquaculture-based Fisheries management programmes if sub-optimal environmental conditions affect the growth and the survival of the introduced captive individuals. This process is difficult to unravel with genetic metrics as it does not affect neutral genetic variation, unless genetic markers directly or indirectly involved in the selection process are used^17,31,32^. Finally, the Baseline genetic information here gathered may help improve the sustainable management of *Mugil cephalus* fisheries by setting the stage for the genetic monitoring of wild populations. Monitoring genetic metrics over time is fundamental to evaluate the impact of supplementation programmes on wild populations, as well as the health, exploitation, recruitment and connectivity patterns of natural stocks^39^.

## Methods

### Ethical approval

The study did not involve endangered or protected species. No specific permissions were required for locations and activities. Capture, non-lethal sampling and experimental protocols followed the principles of laboratory animal care and regulations on animal welfare enforced by national laws (D. Lgs 116/1992 and D. Lgs. 26/2014) and EU Directive 2010/63/EU. No approval was needed by an institutional ethics committee: fin-clips were obtained from fishes that had to be sold in local markets or used to produce roe mullet and were kindly provided by local fishermen.

### Study area

Sardinia island is located in the Western Mediterranean (Fig. 1) and approximately 150 km^2^ are covered by wetlands, which are exploited for fishing and semi-intensive aquaculture of euryhaline fish^11^. In the present study, a small portion of caudal fin was clipped from individuals in four coastal ponds where either semi-intensive aquaculture for sea ranching or traditional fisheries of grey mullet are common practises.

Cabras, Mistras, Porto Pino and Tortolì ponds (Fig. 1) are confined, shallow, non-tidal systems, which differ for extent and physico-chemical characteristics (Supplementary Table S1). Cabras pond (CAB) is a hypertrophic system, in which salinity may drop to < 10 PSU (Practical Salinity Units) in winter and raise to > 30 PSU in summer, depending on the rainfall^43^. Mistras pond (MIS) and Porto Pino salty ponds (PPI) are oligotrophic and hyperhaline systems in which salinity may increase above 40 PSU^44–46^. Tortolì coastal pond (TOR) is a euhaline basin characterised by low eutrophication levels because of a good water exchange with the sea^47^. Salinity ranges between 31 and 38 PSU in winter and summer, respectively^44^.

### Sampling, DNA extraction and PCR protocols

Caudal fin-clips of *M. cephalus* (*N* = 200) were collected during 2013, preserved in absolute ethanol and stored at − 80 °C until DNA isolation. Genomic DNA was purified using the salting-out extraction method^48^, and then stored in TE buffer. DNA quantity and quality were assessed using a fluorimeter (Nanodrop 2000) and diluted if necessary.

Multiplex Polymerase Chain reaction (PCR) protocols were setup to amplify 14 microsatellite markers, which were marked on the 5′ end of the forward primer with the 6-FAM, VIC, NED and PET fluorochromes (Supplementary Table S2). PCR reactions contained 20–30 ng of genomic DNA, 1X reaction buffer (Euroclone), 2 mM MgCl2, 0.250 µM of each dNTP, 0.06–0.14 µM of each primer, 1.25 U of EuroTaq DNA polymerase (Euroclone). PCR reactions were carried out on a MJ DNA Engine PTC-100 thermal cycler under the following conditions: an initial denaturation step at 94 °C for 5 min; 30 cycles of 94 °C for 30 s, annealing temperature of each multiplex for 30 s, 72 °C for 30 s; a final extension of 72 °C for 7 min. After chequing for successful amplicons by electrophoresis on a 2% Agarose gel stained with Ethidium Bromide, 1 μl of PCR product mixed with 9.90 μl of Formamide and 0.10 μl of GeneScan 500 (-250) LIZ size standard (Applied Biosystems) was run on an ABI PRISM 3130xl Genetic Analyser (Applied Biosystems). Microsatellite alleles were scored and binned using GeneMapper v4.0 software package (Applied Biosystems).

### Population genetics analysis

Departure from Hardy–Weinberg proportions (HWP), and linkage disequilibrium (LD) were tested following^49^. First, the Markov chain method (10,000 dememorization steps, 100 batches of 10,000 iterations each) implemented in Genepop 4.7^50^ was used to compute the probability of HWP departures for either heterozygote deficit or heterozygote excess and LD. The binomial likelihood method (LM) was used to combine probabilities across individual tests as it is not affected by small probability values as the Fisher’s exact test is^51^. The joint probability of departure from HWP was computed combining probabilities of single tests by locus or by populations. For LD, single tests were grouped by locus pair or population. If the joint probability within each group was smaller than 0.05, the B–Y method of correction for multiple tests was used to adjust the probability values of single tests^52^. The procedures above were automated running two customised scripts in the R 3.5.2 statistical environment^53^, one of which was designed for carrying out the LM method, while the other was used in Cossu et al.^54^.

The presence of null alleles, stuttering, and large allele dropouts was tested using Micro-checker 2.2.3^55^. The frequency of null alleles was also estimated using FreeNA^56^ to minimise the rate of both false positives and false negatives: only null alleles that reached a frequency > 8% by both methods were deemed as true^57^.

Three methods were used to detect outlier loci. BayeScan 2.1^58^ compares a neutral model with a model that include selection. Setting higher prior odds for the former (threshold = 10), proposal distributions were adjusted using 20 pilot runs of 5000 iterations each and then a simulation was run for 150,000 iterations. Records were sampled every 20 iterations after discarding the first 50,000 iterations. The FDR (False Discovery Rate) was set at 5% to correct for multiple testing. The *LnRH* statistic^59^ assumes reduced levels of genetic diversity within populations for outlier loci. Under neutrality, 95% of neutral loci are expected to show standardised *LnRH* values (mean = 0, standard deviation = 1) between − 1.96 and + 1.96, and between − 2.87 and + 2.87 after correction with the B–Y method. Finally, the FDIST2 approach with a finite island model of migration implemented in Arlequin 3.5.2^60^ was run assuming 100 demes and 100,000 permutations. Probability values were corrected using the B–Y method. Only loci detected as outliers by all methods were deemed as true candidates for selection^61^.

Within population summary statistics, population size reductions and contemporary effective population sizes were assessed following Cossu et al.^54^. The number of alleles (*N*_*A*_), allelic richness (*A*_*R*_), expected and observed heterozygosity (*H*_E_ and *H*_O_) and the inbreeding coefficient (*F*_*IS*_) were computed using diveRsity 1.9.9^62^. Non-parametric pairwise Wilcoxon rank-sum tests were used to assess if *H*_E_ and *A*_R_ were different between population pairs. Signatures of recent population declines were assessed using the heterozygosity excess test implemented in BOTTLENECK 1.2^63^. The contemporary effective population size (*N*_*e*_) was estimated using the linkage disequilibrium (LD) method implemented in NeEstimator V2.1^64^, setting the minor allele frequency to 0.02^65^.

Genetic differentiation amongst populations was assessed using diveRsity to compute the Weir and Cockerham’s *F*_ST_ estimator *θ*^66^ and Jost’s *D*_EST_^67^ index: means and confidence intervals were assessed carrying out 10,000 bootstrap replicates. Exact G tests implemented in Genepop 4.7 were used to compute the probability values of population differentiation. Probabilities of pairwise multiple tests were adjusted applying the B–Y method^52^.

POWSIM V1.2^68^ was used to estimate the statistical power of detecting population differentiation. Pseudo-datasets with the same number of populations (*N*), loci, alleles and population sampling size (*S*) as the real dataset were created. Different levels of genetic differentiation (*F*_ST_ = 0.00–0.01) were simulated assuming constant population effective size (*N*_e_ = 2000), no migration and varying the time since divergence. The statistical power was evaluated computing the fraction of both Chi-square and Fisher’s exact tests that successfully detected population differentiation out of 1000 replicates for each simulation.

Kinship analyses were carried out using the method of Blouin et al.^69^, implemented in DEmerelate 0.9.3^70^. The method tests for an overabundance of closely related individuals by comparing the proportions of full- and half-siblings observed in empirical populations with those expected in randomised reference populations. Furthermore, to assess the uncertainty in sibship reconstruction^36^, relationships among individuals were cross-chequed using ML-Relate^71^.

Genetic structure was investigated using the Bayesian model-based clustering implemented in structure 2.3.4^72^. Only genetic information was used to group individuals into clusters that minimise Hardy–Weinberg–Linkage disequilibria. Simulations were run using the admixture model with correlated allele frequencies^73^ and varying the number of clusters (*K* = 1–4). For each *K*, 10 independent runs were performed, each consisting of 100,000 iterations following a burn-in period of equal length. The mean posterior probability (*lnP*(*D*)) and its standard deviation (*SD*) were computed using the Pophelper 1.0.10 web app^74^. In addition to model-based Bayesian clustering, a Discriminant Analysis of Principal Components (DAPC), which does not rely upon a population genetic model^75^, was carried out in the adegenet R package^76^. DAPC aimed at visualizing differences among ponds as prior groups. The number of retained principal components was optimised using a cross-validation procedure to avoid overfitting of the data.

## Supplementary Information

Supplementary Tables.

Supplementary Figures.

## Data Availability

The dataset generated and analysed during the current study is available in the figshare repository, 10.6084/m9.figshare.12594374.

## Code availability

Custom R codes used for data analysis are available in the figshare repository, 10.6084/m9.figshare.12594374 and in the DRYAD repository, https://doi.org/10.5061/dryad.dm97908.
