# Supplementary material for: Genetic patterns in Mugil cephalus and implications for fisheries and aquaculture management
Source: Sci Rep. 2021 Feb 3;11:2887. doi: 10.1038/s41598-021-82515-7 (PMC7859195; doi:10.1038/s41598-021-82515-7)
Supplement: Supplementary file 1 — Supplementary Tables. [file 41598_2021_82515_MOESM1_ESM.pdf]

Online Resource 1

P. Cossu[1,2], L. Mura[3], F. Scarpa[1,2], T. Lai[1], D. Sanna[1,4], I. Azzena[2], N. Fois[3], M. Casu[1,2]

Genetic patterns in *Mugil cephalus* and implications for fisheries and aquaculture management

[1]Department of Sciences for Nature and Environmental Resources, University of Sassari, Sassari, Italy

[2] Department of Veterinary Medicine, University of Sassari, Sassari, Italy

[3]Dipartimento per la Ricerca nelle Produzioni Animali, Agris Sardegna, Olmedo (SS), Italy

[4] Department of Biomedical Sciences, University of Sassari, Sassari, Italy

Corresponding author:

Piero Cossu

Department of Veterinary Medicine

University of Sassari

Sassari (SS), Italy

picossu@uniss.it

ORCID: 0000-0002-8946-8261

Table S1 Characteristics of the four coastal ponds. CAB = Cabras pond; MIS = Mistras pond; PPI = Porto Pino ponds; TOR = Tortolì pond

	CAB	MIS	PPI	TOR
Tipology	Non tidal	Non tidal	Non tidal	Non tidal
Area (Km^2)	22.3	4.50	5	2.89
Mean Depth (m)	1.6	0.5	0.7	1.10
Salinity[(a)]	Mesohaline	Hyperhaline	Hyperhaline	Euhaline
Trophic status	Hypertrophic	Oligotrophic	Oligotrophic	Eutrophic

(a) Based on mean annual salinity: classification of transitional waters follows the European Water Framework Directive 2000/60/EC: <0.5 ‰, Freshwater; <0.5 ‰ to <5 ‰, Oligohaline; 5 ‰ to 18 ‰, Mesohaline; 18 ‰ to <30 ‰, Polyhaline; 30 ‰ to <40 ‰, Euhaline; >40 ‰, Hyperhaline

Table S2 Microsatellite loci used in this study. T_A = Annealing temperature of each primer (°C); C = primer concentration (µM) in each PCR reaction; N_A = Number of alleles

Locus	Primer sequence	*Fluorochrome*	T_A	*C*	*Multiplex*	N_A	Allele range size
Mce22	F: ACGCATTGAGCAGACATGAACT<br>R: TGACCCTACGTACGTATCAG	6-FAM	50	0.08	A	5	116-124
Mce7	F: GCCCCGAGATGGAGAAGA<br>R: AAGATGGAACAAGGCAAAGAG	PET	50	0.12	A	20	174-248
Mce14	F: TAATACGATGTGCTTTGTCCC<br>R: CATAACCTCCACCTGCTGAC	6-FAM	50	0.12	A	14	154-190
Mce6	F: GAGGAGGCTCGGAGGATT<br>R: CGGGGCTTGTGACAGTTT	VIC	50	0.12	A	16	180-214
Mce11	F: ATTAGCCAGGGCCACCAG<br>R: CAGAAGCCAAAAGGACGG	VIC	58	0.06	B	4	165-171
Mce24	F: AAAGTCTGAATGGAGCGCAG<br>R: TCCTGAATCTCTGGTGACAGC	PET	58	0.09	B	22	119-179
Mce25	F: TCGGCATGTATATGAAAGCAC<br>R: ACATAACTCTGCCACTGCTTG	NED	58	0.12	B	34	123-241
Mce3	F: GGAGGACTAGGATTTCGTG<br>R: CTTATCCCTGACCTTCTCAT	6-FAM	50	0.08		10	137-177
Mcs15A M	F: GAGCCAAACTGGTCACATGAAAGAGA<br>R: ACTTTCAGTGCAGCGCCCAGTGTT	VIC	60	0.14	C	25	193-289
Mcs15C M	F: GGATTAGTGGCGGACTTCTGTGAA<br>R: CTCTTTCAATTATGTCAGTGGTATGGCTTC	NED	60	0.12	C	22	155- 61
Mcs16D M	F: CAAATACTAGATGCTTTGCACACCCTTAC<br>R: GGCCTACAGATTTCCTTCTTTATGCTAGGC	PET	60	0.11	C	12	153-203
Mcs1EH	F: ACCGGGCTTTAGGCTGTTGGTCA<br>R: TGAGACACATCCCATCACTGCCTACG	6-FAM	60	0.10	C	19	269-341
Mcs2DM	F: CATGGGCATCTTTATCGCTCTCAA<br>R: CAACTTAATTTCCCTTCGGGGATGA	PET	56	0.12	D	31	182-278
Mcs2FH	F: GCAGTGAAATAAACAGGCACCTCTCA<br>R: TGTGGGGTATGAAGTCCTCTTGCTC	6-FAM	56	0.06	D	26	187-267

Table S3. Allele frequencies of the 14 loci used in the present study. Private alleles are marked in bold. CAB = Cabras pond; MIS = Mistras pond; PPI = Porto Pino ponds; TOR = Tortolì pond

Locus	Allele size	CAB	MIS	PPI	TOR
Mce22					
	116	0.029	0.088	0.149	0.070
	118	0.788	0.794	0.628	0.680
	120	0.154	0.103	0.213	0.220
	122	0.019	0.015		0.010
	124	0.010		0.011	0.020
Mce7					
	174		0.015	0.010	0.010
	176	0.010	0.015	0.010	
	184	0.010			
	188		0.044	0.010	0.060
	190	0.644	0.574	0.625	0.640
	192	0.058	0.059	0.083	0.030
	194	0.154	0.162	0.156	0.170
	196	0.038	0.029	0.021	0.030
	197				0.010
	207			**0.010**	
	220	0.019			
	222	0.010			0.020
	224	0.010	0.029		
	226	0.010		**0.010**	
	230	0.010	0.015		
	236	0.019	0.044	0.031	0.020
	238		0.015		
	239			**0.021**	
	242	0.010		0.010	
	247				**0.010**
Mce14					
	154		**0.015**		

Locus	Allele size	CAB	MIS	PPI	TOR
	156	0.471	0.412	0.333	0.460
	158		0.044		0.010
	162	0.067	0.103	0.083	0.080
	164	0.173	0.132	0.208	0.170
	166	0.038	0.074	0.104	0.070
	168	**0.010**			
	170	0.019	0.015	0.010	0.010
	172	0.038	0.044	0.063	0.030
	176	0.115	0.088	0.125	0.100
	178			**0.010**	
	180		0.044	0.031	0.030
	188	0.058	0.029	0.021	0.030
	190	0.010		0.010	0.010
Mce6					
	180				**0.010**
	182	0.250	0.265	0.213	0.210
	184				0.020
	188	0.038	0.015	0.053	0.040
	190				0.010
	192	0.365	0.397	0.394	0.450
	194	0.077	0.074	0.085	0.050
	196	0.019		0.011	0.010
	198	0.010		0.011	0.020
	200	0.173	0.206	0.128	0.080
	202	0.029	0.029	0.085	0.060
	204	0.010	0.015		0.010
	206			0.011	0.010
	208	**0.010**			
	212	**0.010**			
	213	**0.010**			

Locus	Allele size	CAB	MIS	PPI	TOR
	214			0.011	0.020
Mce11					
	157			**0.010**	
	161			**0.010**	
	165	0.038	0.029	0.042	0.040
	167	0.769	0.779	0.698	0.740
	169	0.183	0.191	0.229	0.220
	171	0.010		0.010	
Mce24					
	119	0.048	0.029	0.031	0.020
	121	0.010	0.015	0.021	0.010
	125	0.058	0.015	0.042	0.030
	127			**0.010**	
	129	0.067	0.074	0.094	0.080
	131	0.019	0.015		0.010
	133	0.029	0.015	0.042	0.010
	135	0.048	0.029	0.042	0.040
	137	0.154	0.162	0.125	0.140
	141	0.154	0.176	0.135	0.160
	143	0.115	0.176	0.115	0.150
	145		0.015	0.010	0.010
	147	0.029	0.059	0.010	0.040
	149	0.048	0.088	0.094	0.090
	151	0.077	0.044	0.052	0.090
	153	0.029	0.029	0.063	0.020
	155	0.029		0.052	0.060
	157	0.038	0.029	0.031	
	159		0.015	0.010	
	161	0.010	0.015		0.030
	163	**0.010**			

Locus	Allele size	CAB	MIS	PPI	TOR
	173	0.029		0.010	0.010
	179			**0.010**	
Mce25					
	123	0.029	0.015	0.043	0.030
	125	0.077	0.059	0.022	0.040
	127	0.019		0.011	0.030
	129	**0.010**			
	131	0.231	0.382	0.163	0.200
	133	0.288	0.265	0.348	0.320
	135	0.135	0.074	0.174	0.110
	137			**0.011**	
	139	0.067	0.059	0.033	0.060
	141			0.022	0.020
	143		0.015		0.040
	145			0.011	0.030
	153		**0.015**		
	159	**0.010**			
	165			**0.011**	
	167				**0.010**
	169			**0.011**	
	171	0.010	0.015	0.011	0.020
	173	0.010		0.011	0.020
	175		0.044	0.022	0.010
	177	0.029	0.029	0.011	
	179	**0.010**			
	181	0.010			0.010
	183			0.011	0.020
	191	0.010			0.010
	197		**0.015**		
	199			**0.011**	

Locus	Allele size	CAB	MIS	PPI	TOR
	203			**0.011**	
	205	**0.010**			
	209		**0.015**		
	211			0.011	0.010
	213				**0.010**
	217	**0.010**			
	219	**0.010**			
	225	0.029		0.011	
	231			**0.011**	
	235			**0.011**	
	241			**0.011**	
Mce3					
	137		**0.015**		
	139	0.010		0.021	
	145		**0.030**		
	147	0.096	0.106	0.063	0.100
	149	0.808	0.803	0.844	0.800
	151	0.077	0.030	0.052	0.070
	153				0.020
	155	**0.010**			
	157			0.010	0.010
	159			**0.010**	
	177		**0.015**		
Mcs15AM					
	193			**0.021**	
	197	0.029			0.010
	201	0.019			0.010
	205			0.011	0.020
	209	0.019	0.029	0.032	0.010
	213		0.074	0.043	0.010

Locus	Allele size	CAB	MIS	PPI	TOR
	217	0.058	0.059	0.032	0.020
	221	0.048	0.044	0.043	0.092
	225	0.058	0.059	0.043	0.020
	229	0.058	0.029	0.074	0.143
	233	0.048	0.059	0.117	0.051
	237	0.067	0.191	0.128	0.102
	241	0.087	0.059	0.085	0.102
	245	0.144	0.044	0.149	0.102
	249	0.096	0.074	0.043	0.041
	253	0.058	0.029	0.043	0.041
	257	0.038	0.074	0.032	0.041
	261	0.058	0.074	0.043	0.041
	265	0.010	0.015	0.053	0.051
	269	0.029	0.044		0.031
	273	0.019	0.029		0.020
	277	0.029	0.015		
	281			0.011	0.010
	285	0.010			0.020
	289	0.010			0.010
	333	**0.010**			
Mcs15CM					
	155	0.298	0.250	0.260	0.150
	159	0.038		0.031	0.030
	163	0.010	0.029	0.021	
	167			**0.010**	
	171		0.015	0.010	0.010
	175	0.010			0.020
	179	0.010	0.015	0.052	0.020
	183	0.038	0.015	0.042	0.030
	187	0.038	0.015	0.052	0.060

Locus	Allele size	CAB	MIS	PPI	TOR
	191	0.106	0.088	0.083	0.120
	195	0.048	0.059	0.063	0.080
	199	0.077	0.044	0.104	0.080
	203	0.087	0.103	0.031	0.110
	207	0.125	0.162	0.125	0.040
	211	0.058	0.074	0.031	0.060
	215	0.019	0.044	0.031	0.070
	219		0.059	0.010	0.040
	223	0.038	0.015	0.021	0.040
	227				0.010
	231		0.015	0.021	0.010
	245				0.010
	261				0.010
Mcs16DM					
	145			**0.010**	
	153	0.240	0.279	0.125	0.210
	157			**0.010**	
	161	0.048	0.029	0.104	0.090
	165	0.019	0.088	0.052	0.090
	169	0.010	0.059	0.010	
	173	0.231	0.162	0.240	0.230
	177	0.269	0.294	0.240	0.250
	181	0.106	0.044	0.156	0.080
	185	0.038	0.015	0.010	0.020
	189	0.019		0.021	0.010
	197		0.015		0.010
	201		0.015	0.021	0.010
	203	**0.019**			
Mcs1EH					
	269	0.010	0.015	0.011	0.010

Locus	Allele size	CAB	MIS	PPI	TOR
	273		0.015	0.043	
	277	0.173	0.103	0.096	0.100
	281	0.106	0.176	0.128	0.180
	285	0.058	0.103	0.085	0.050
	289	0.087	0.176	0.096	0.060
	293	0.029	0.015	0.064	0.030
	297	0.096	0.132	0.128	0.160
	301	0.087	0.059	0.053	0.060
	305	0.067	0.044	0.053	0.060
	309	0.106	0.044	0.074	0.070
	313	0.010	0.015	0.043	0.060
	317	0.029	0.029	0.032	0.070
	321	0.038	0.015	0.021	0.040
	325	0.067		0.021	0.040
	329	0.029	0.029	0.011	0.010
	333	0.010		0.011	
	337			0.011	
	341		0.029	0.021	
McS2DM					
	182	**0.010**			
	194	0.010		0.010	0.020
	198			0.010	0.020
	204		0.015	0.010	
	206	0.019	0.029	0.042	0.030
	210	0.029	0.059	0.052	0.110
	212		0.015	0.010	
	214	0.087	0.059	0.083	0.070
	216			0.010	0.010
	218	0.058	0.044	0.031	0.060
	220		0.015	0.031	

Locus	Allele size	CAB	MIS	PPI	TOR
	222	0.029	0.059	0.031	0.090
	226	0.087	0.088		0.020
	228		0.015	0.021	
	230	0.106	0.132	0.042	0.050
	232			**0.031**	
	234	0.125	0.118	0.052	0.090
	236			**0.031**	
	238	0.115	0.044	0.021	0.060
	240			**0.010**	
	242	0.048	0.044	0.094	0.090
	244	0.010	0.015	0.073	0.010
	246	0.067	0.074	0.021	0.080
	248			**0.021**	
	250	0.048	0.059	0.031	0.030
	252	0.010		0.031	
	254	0.058	0.015	0.052	0.020
	255			**0.010**	
	258	0.029	0.015	0.042	0.030
	262	0.010		0.021	0.020
	264	0.010		0.010	0.050
	266	0.019	0.044	0.031	0.010
	270	0.010	0.015	0.021	0.030
	274		0.029	0.010	
	278	**0.010**			
Mcs2FH					
	187	0.010	0.015		0.020
	193	0.096	0.118	0.073	0.110
	195	0.308	0.250	0.302	0.250
	197	0.010	0.044	0.052	0.010
	199	0.029	0.103	0.063	0.050

Locus	Allele size	CAB	MIS	PPI	TOR
	201	0.038		0.010	0.020
	203	**0.038**			
	205			0.021	0.010
	207	0.019		0.021	0.020
	211	0.010		0.042	0.010
	215	0.038	0.015	0.010	0.030
	219	0.019	0.059	0.010	0.020
	221			**0.021**	
	223	0.038		0.042	0.110
	227	0.058	0.029	0.010	0.070
	229			**0.021**	
	231	0.077	0.074	0.063	0.070
	233			**0.021**	
	235	0.058	0.059	0.063	0.060
	237		0.015	0.021	
	239	0.019	0.044	0.063	0.030
	241		0.015	0.010	
	243	0.010	0.015	0.031	0.020
	245		**0.015**		
	247	0.058	0.044	0.021	0.030
	251	0.019	0.059	0.010	0.040
	255	0.019	0.015		0.020
	259	**0.019**			
	263	**0.010**			
	267		**0.015**		

Table S4 Summary statistics of within population genetic variation for each locus and population of *Mugil cephalus*. A = Number of alleles; A_R = Allelic richness; H_O = Observed heterozygosity; H_E = Expected heterozygosity; F_{IS} = Inbreeding coefficient; HWP_D = probability of departure from Hardy-Weinberg proportions because of heterozygote deficit; HWP_E = probability of departure from Hardy-Weinberg proportions because of heterozygote excess; F_{IS} Low = 95% confidence interval lower limit of inbreeding coefficient; F_{IS} High = 95% confidence interval upper limit of inbreeding coefficient. P-values with P < 0.05 are outlined in bold. For population labels see Table S3

	Mce7	Mce14	Mce6	Mce11	Mce24	Mcs15AM	Mcs15CM	Mcs1EH	Mcs2DM	Mcs2FH
CAB										
A	13	10	12	4	19	22	15	16	23	22
A_R	8.74	8.48	8.94	3.42	16.37	18.37	12.9	13.88	17.93	17.33
H_O	0.58	0.75	0.79	0.42	0.87	0.92	0.81	0.92	0.88	0.92
H_E	0.56	0.72	0.76	0.37	0.91	0.93	0.86	0.91	0.93	0.87
HWP_D	0.66	0.24	0.43	0.87	0.20	0.12	0.45	0.21	0.23	0.95
HWP_E	0.42	0.76	0.57	0.22	0.81	0.88	0.55	0.79	0.76	0.10
F_{IS}	-0.039	-0.037	-0.031	-0.133	0.051	0.009	0.059	-0.018	0.044	-0.060
F_{IS}_Low	-0.176	-0.170	-0.162	-0.283	-0.038	-0.063	-0.034	-0.093	-0.040	-0.123
F_{IS}_High	0.110	0.101	0.112	0.071	0.153	0.094	0.166	0.071	0.139	0.006
MIS										
A	11	11	7	3	18	18	16	16	22	19
A_R	9.14	10	6.15	2.89	14.87	16.52	13.59	13.71	18.7	15.8
H_O	0.65	0.85	0.79	0.38	0.88	0.91	0.97	0.79	0.97	0.88
H_E	0.63	0.78	0.72	0.36	0.89	0.92	0.87	0.89	0.93	0.89
HWP_D	0.16	0.94	0.72	0.76	0.06	0.32	0.98	0.10	0.79	0.09
HWP_E	0.85	0.12	0.27	0.50	0.93	0.69	0.05	0.90	0.28	0.91
F_{IS}	-0.019	-0.091	-0.098	-0.077	0.006	0.007	-0.110	0.106	-0.043	0.007
F_{IS}_Low	-0.186	-0.200	-0.287	-0.295	-0.116	-0.078	-0.165	-0.036	-0.091	-0.115
F_{IS}_High	0.164	0.021	0.098	0.222	0.143	0.111	-0.044	0.258	0.025	0.142
PPI										
A	12	11	10	6	20	18	18	19	32	23
A_R	8.5	9.18	7.99	4.47	16.22	15.98	15.24	16.04	24.86	18.11
H_O	0.58	0.88	0.79	0.5	0.88	0.94	0.85	0.98	0.96	0.98
H_E	0.58	0.81	0.77	0.46	0.92	0.92	0.88	0.92	0.95	0.88
HWP_D	0.29	0.70	0.40	0.84	**0.02**	0.22	0.23	0.98	0.24	0.99
HWP_E	0.74	0.31	0.61	0.20	0.98	0.79	0.77	0.07	0.79	**0.01**
F_{IS}	-0.014	-0.085	-0.028	-0.091	0.047	-0.020	0.032	-0.064	-0.004	-0.117
F_{IS}_Low	-0.178	-0.186	-0.160	-0.277	-0.050	-0.093	-0.060	-0.100	-0.055	-0.175
F_{IS}_High	0.169	0.029	0.114	0.108	0.157	0.069	0.134	-0.013	0.063	-0.062
TOR										
A	10	11	14	3	18	23	20	15	22	20
A_R	7.72	9.15	10.33	2.96	14.59	18.35	16.51	13.65	18.66	16.4
H_O	0.6	0.82	0.66	0.36	0.88	0.9	0.9	0.88	0.94	0.94
H_E	0.55	0.74	0.74	0.4	0.9	0.92	0.92	0.9	0.93	0.89

HWP_D	0.93	0.96	**<0.01**	0.26	0.29	0.19	0.26	0.30	0.57	0.54
HWP_E	0.11	0.05	0.99	0.78	0.71	0.82	0.74	0.70	0.44	0.46
F_{IS}	-0.081	-0.115	0.105	0.105	0.022	0.029	0.020	0.023	-0.006	-0.056
F_{IS}_Low	-0.200	-0.223	-0.041	-0.129	-0.069	-0.052	-0.062	-0.064	-0.067	-0.130
F_{IS}_High	0.042	-0.002	0.256	0.360	0.126	0.122	0.116	0.125	0.066	0.030

Table S5. Estimated null allele frequencies for each locus based on Brookfield's (1996) and Dempster's (1977) methods. Null allele frequencies >0.08 are outlined in bold. For population labels see Table S3

	CAB		MIS		PPI		TOR	
	Brookfield	Dempster	Brookfield	Dempster	Brookfield	Dempster	Brookfield	Dempster
Mce22	0.034	0.045	**0.085**	**0.116**	**0.122**	0.060	0.003	
Mce7				0.013				
Mce14		0.006						
Mce6					0.062		0.045	0.045
Mce11							0.030	0.017
Mce24	0.025	0.012	0.003	0.022	0.022	0.030	0.010	
Mce25	**0.118**	**0.116**			**0.163**	0.047	**0.084**	**0.092**
Mce3	**0.105**	**0.126**	**0.210**	**0.141**	0.024	0.036	0.079	**0.116**
Mcs15AM	0.004	0.011	0.003		0.042	0.005	0.058	0.004
Mcs15CM	0.027				0.015	0.005	0.009	0.003
Mcs16DM								
Mcs1EH		0.005	0.050	0.037	0.027		0.011	0.004
Mcs2DM	0.021	0.003				0.002		
Mcs2FH			0.003	0.020				

Table S6 Pairwise population differentiation estimated using Jost's D_{EST}. Observed values are reported below the diagonal. Lower and upper limits of 95% confidence intervals based on 10'000 bootstraps are reported within brackets above the diagonal. Values outlined in bold indicate population pairs showing significant genetic divergence based on the Fisher's exact test for genetic homogeneity of genotype frequencies. Due to multiple testing, probability values were adjusted using the False Discovery Rate (FDR) method (Benjamini and Yekutieli 2001). For population labels see Table S3

	CAB	MIS	PPI	TOR
CAB	—	(-0.0220, 0.0323)	(-0.0178, 0.0250)	(-0.0201, 0.0291)
MIS	0.000	—	(-0.0253, 0.0360)	(-0.0269, 0.0367)
PPI	0.0004	0.0002	—	(-0.0241, 0.0329)
TOR	0.0015	0.0011	0.0002	—

Table S7 Bayesian clustering results. K = number of tested genetic clusters; mean *ln*(PD) = natural logarithm of posterior probability of data averaged over runs; SD *ln*(PD) = Standard Deviation of natural logarithm of posterior probability of data; Min *ln*(PD) minimum value of natural logarithm of posterior probability of data; Max *ln*(PD) maximum value of natural logarithm of posterior probability of data. The most likely clustering solution is outlined in bold

K	Number of runs	Mean *ln*(PD)	SD ln(PD)	Min ln(PD)	Max ln(PD)
**1**	**10**	**-8370.51**	**1.22**	**-8373.2**	**-8369.1**
2	10	-8691.91	196.03	-9160.2	-8370
3	10	-9534.12	682.08	-10919.7	-8593.7
4	10	-9296.91	449.83	-10250.1	-8813.2

Table S8 Proportion of full-sibling and half-sibling dyads estimated in each population using the genotype sharing method implemented in Demerelate and the maximum likelihood method implemented in ML-Relate softwares, respectively

	Full-siblings (%)		Half-siblings (%)	
	Demerelate	*ML-Relate*	*Demerelate*	*ML-Relate*
CAB	0.98	0.38	33.79	7.16
MIS	1.29	1.08	23.87	4.73
PPI	0.35	0.27	24.29	5.59
TOR	0.49	0.33	31.02	6.61
