# Supplementary material for: Genetic patterns in Mugil cephalus and implications for fisheries and aquaculture management
Source: Sci Rep. 2021 Feb 3;11:2887. doi: 10.1038/s41598-021-82515-7 (PMC7859195; doi:10.1038/s41598-021-82515-7)
Supplement: Supplementary file 2 — Supplementary Figures. [file 41598_2021_82515_MOESM2_ESM.pdf]

P. Cossu[1,2], L. Mura[3], F. Scarpa[1,2], T. Lai[1], D. Sanna[1,4], I. Azzena[2], N. Fois[3], M. Casu[1,2]

Genetic patterns in *Mugil cephalus* and implications for fisheries and aquaculture management

[1]Department of Sciences for Nature and Environmental Resources, University of Sassari, Sassari, Italy

[2] Department of Veterinary Medicine, University of Sassari, Sassari, Italy

[3]Dipartimento per la Ricerca nelle Produzioni Animali, Agris Sardegna, Olmedo (SS), Italy

[4] Department of Biomedical Sciences, University of Sassari, Sassari, Italy

Corresponding author:

Piero Cossu

Department of Veterinary Medicine

University of Sassari

Sassari (SS), Italy

picossu@uniss.it

ORCID: 0000-0002-8946-8261

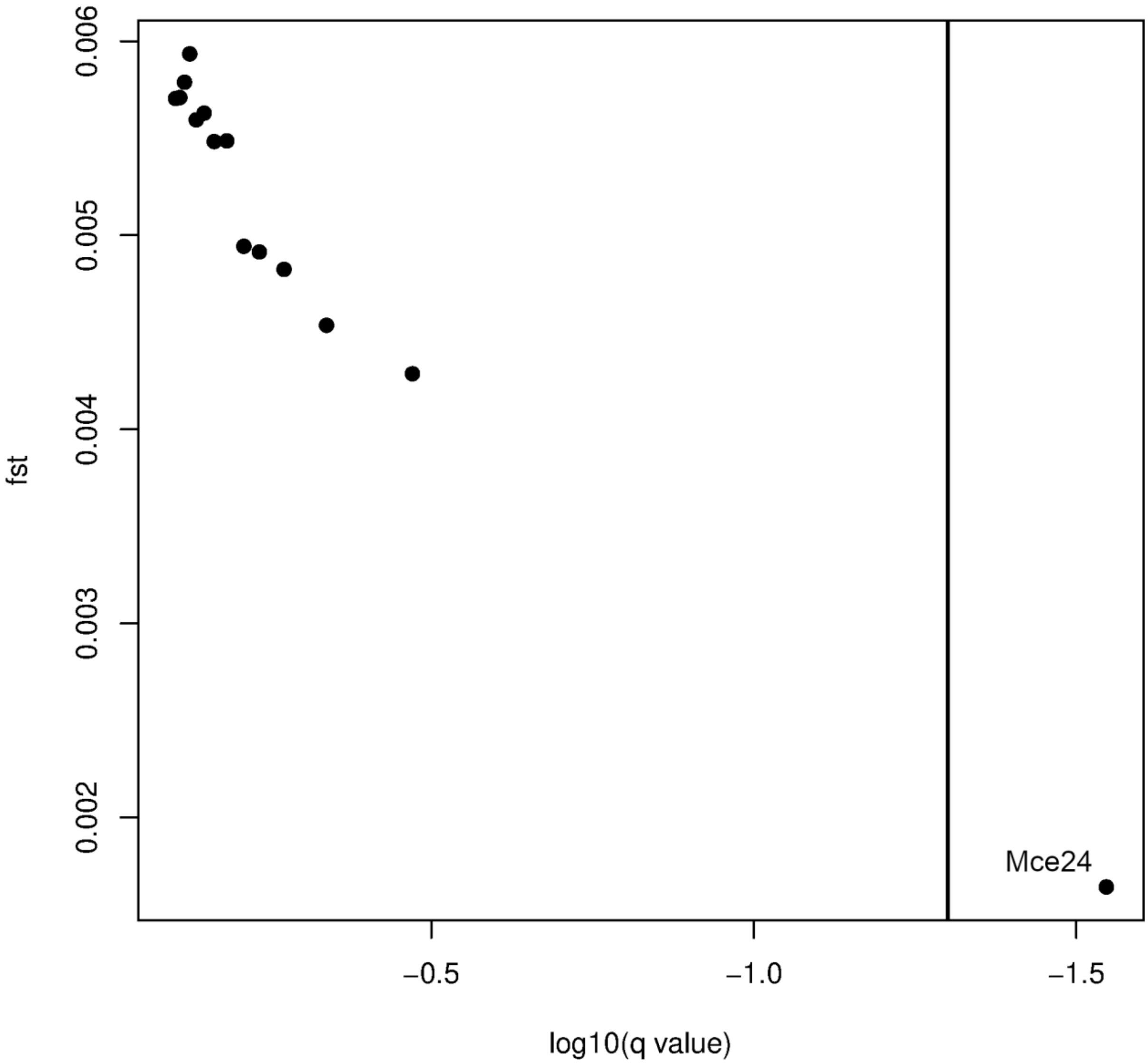

Fig. S1 Detection of outlier loci with the Bayesian method implemented in Bayescan. Points represent the F_{ST} values for the 14 loci used in the present study. The vertical black line corresponds to the threshold above which loci are deemed as outlier for divergent (right of the black line, on the top) or balancing (right of the black line, on the bottom) selection. Outlier loci are annotated by the locus name. This figure was created using the R 3.5.2 (https://www.r-project.org/)

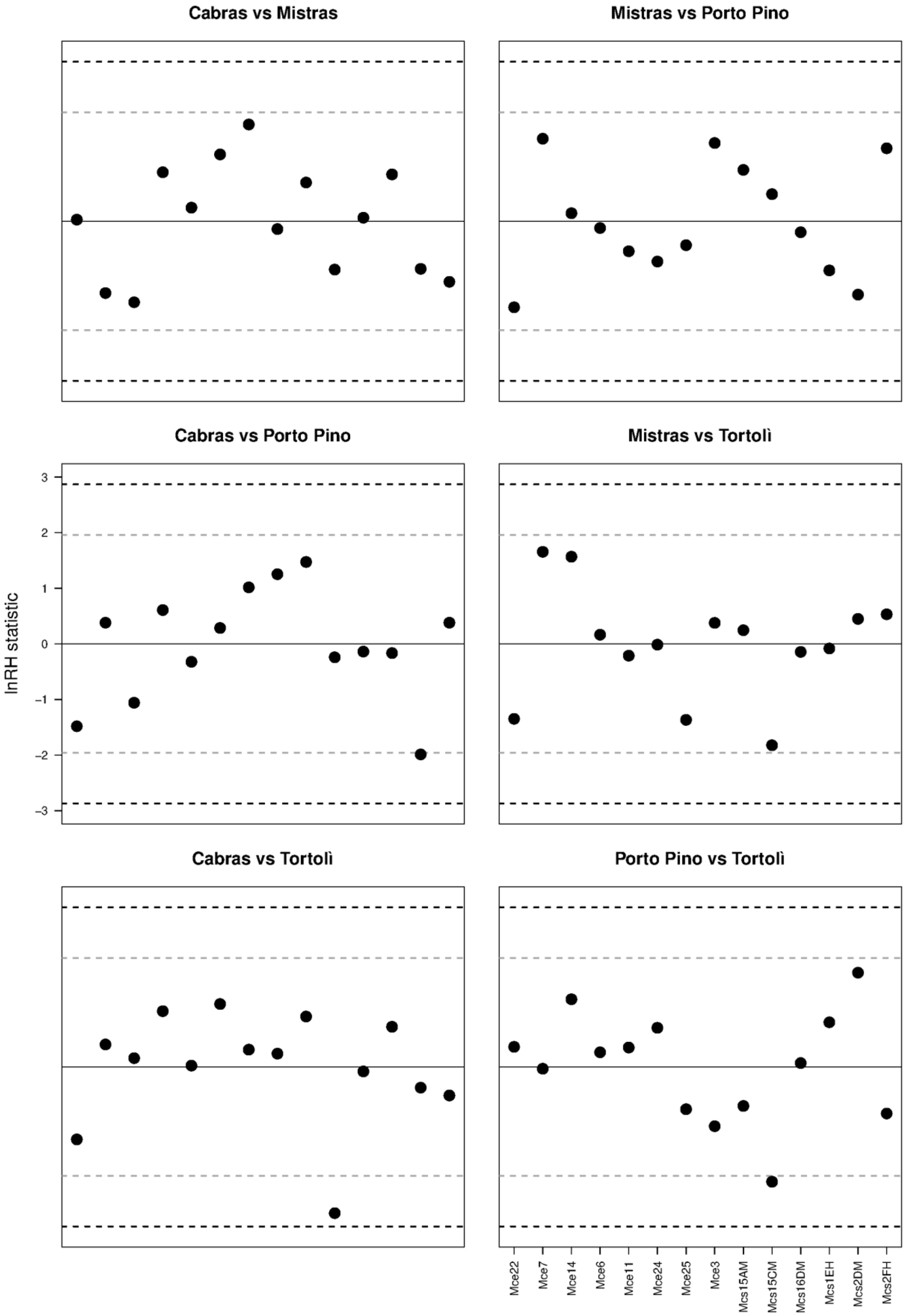

Fig. S2 Detection of outlier loci based on the standardized *Ln*RH statistic. Gray dashed lines mark the lower and upper bounds (±1.96) of the 95% confidence interval (CI) of the expected distribution of neutral loci; black dashed lines mark the lower and upper bounds (±2.87) of the CI adjusted by the False Discovery Rate (FDR) method (Benjamini & Yekutieli 2001. This figure was created using the R 3.5.2 (https://www.r-project.org/) and then modified using the free software GIMP 2.10.2 (https://www.gimp.org/)

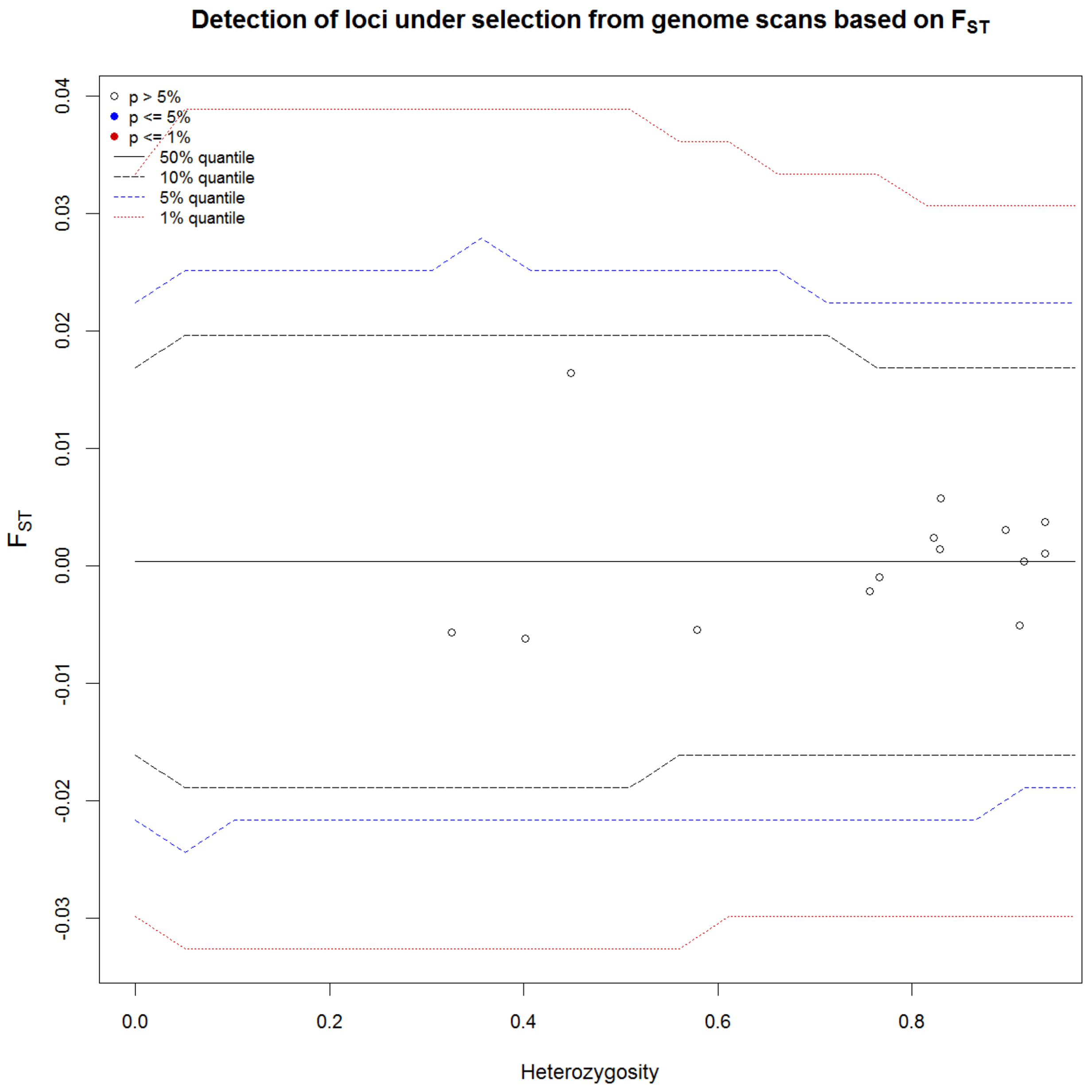

Fig. S3 Detection of outlier loci with the FDIST2 method implemented in Arlequin. Circles represent the F_{ST} values for the 14 loci used in the present study; solid and dashed lines outline the quantiles delimiting the distribution of F_{ST} values expected at selective neutrality. Loci are deemed as outliers if their F_{ST} values fall above (divergent selection) or below (balancing selection) the expected neutral distribution. This figure was created using the R 3.5.2 (https://www.r-project.org/)

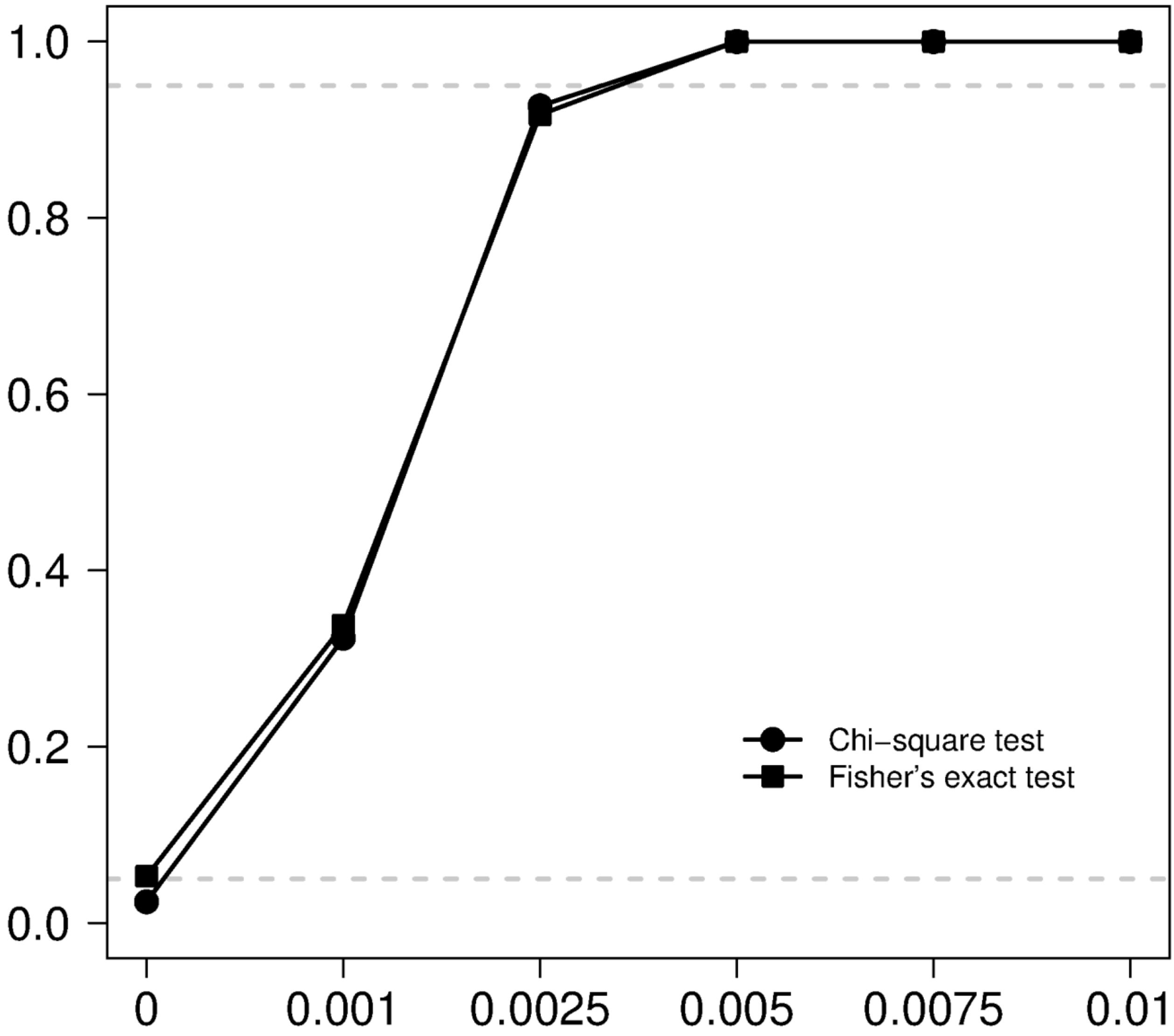

Fig. S4 Power of detecting population differentiation. Simulations were carried out assuming a population size of N_e = 2'000, whereas number of populations, their sample size and the number of alleles as the real data. Starting allele frequencies were computed by pooling all populations. F_{ST} values (x- axis) were obtained by increasing the number of generations of genetic drift (*t* =0, 4, 10, 20, 30, 40). The proportion of significances (y-axis) corresponds to α error when F_{ST} = 0 and to power when F_{ST} > 0. Gray dashed lines correspond to a proportion of significances = 0.05 and 0.95, the former being the threshold for the expected α error. This figure was created using the R 3.5.2 (https://www.r-project.org/)

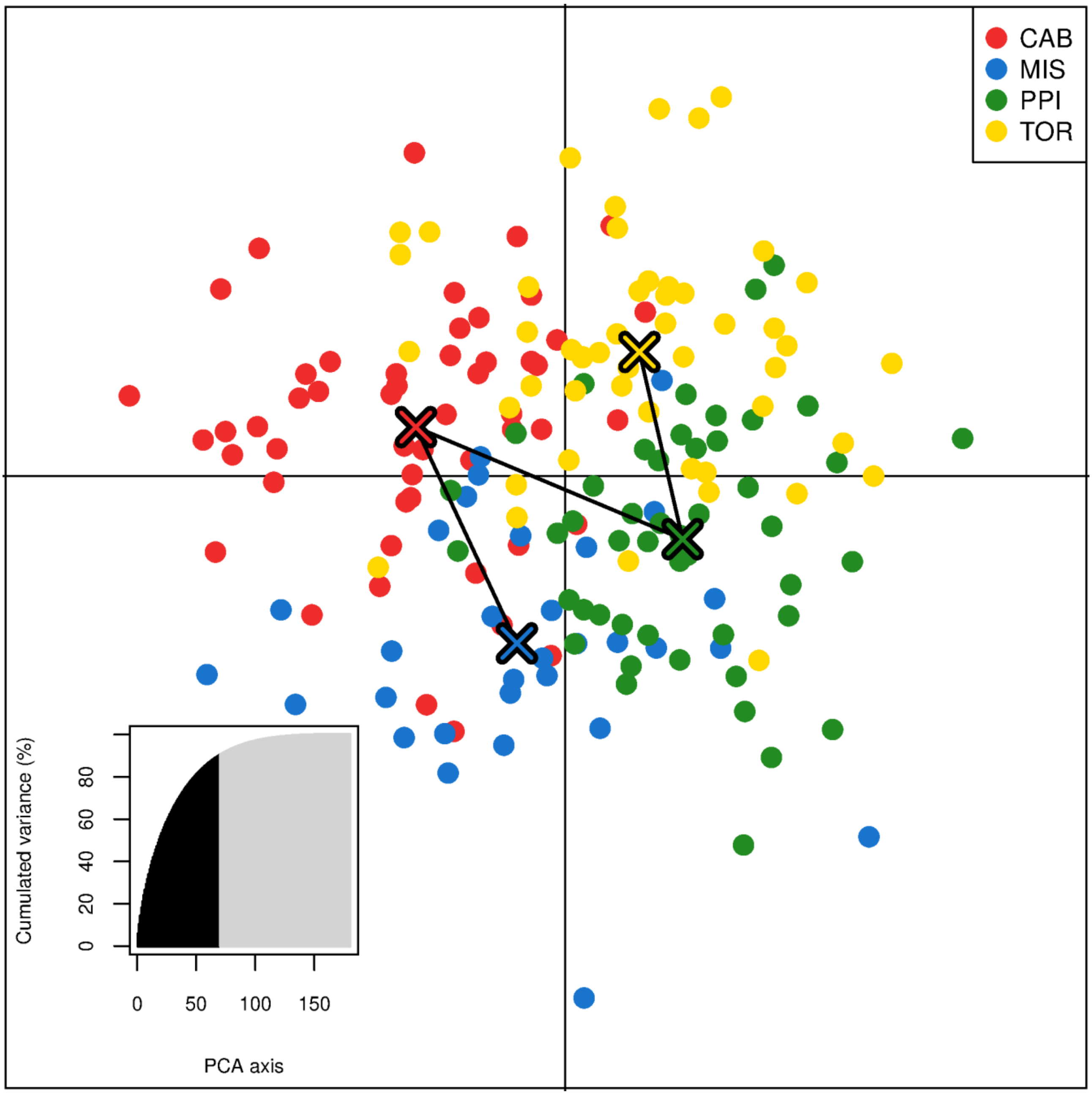

Fig. S5 Scatterplot of the first two discriminant functions of Discriminant Analysis of Principal Components (DAPC). Using ponds as prior groups, 70 principal components (PC) were retained based on the Minimum Root Mean Squared Error (RMSE) resulting from the cross-validation procedure. The inset depicts the cumulative fraction of total variance described by the retained PCs. The solid black lines represent the minimum spanning network (MSN) connecting the centroids of each group of points. This figure was created using the R 3.5.2 (https://www.r-project.org/)
